# Cultivated Non-Native Vascular Plants of Kazakhstan: Taxonomic Composition, Regional Distribution and Establishment Status

**DOI:** 10.3390/plants15213433

**Published:** 2026-11-04

**Authors:** Serik Kubentayev, Daniyar Alibekov, Anar Myrzagaliyeva, Margarita Ishmuratova, Saule Baibossynova, Alexander N. Sennikov

**Affiliations:** 1 Laboratory “NatureLab”, Higher School of Natural Sciences, Astana International University, Astana 010000, Kazakhstan; kubserik@mail.ru (S.K.); an.myrzagaliyeva@gmail.com (A.M.); 2 Astana Botanical Garden, Astana 010000, Kazakhstan; dansilvaforest@mail.ru (D.A.); baisaule-m@mail.ru (S.B.); 3 Faculty of Biology and Geography, Karaganda National Research University Named After Academician E.A. Buketov, Karaganda 100028, Kazakhstan; margarita.ishmur@mail.ru; 4 Botanical Museum, Finnish Museum of Natural History, University of Helsinki, 00014 Helsinki, Finland

**Keywords:** alien plants, Central Asia, cultivated flora, escape from cultivation, horticulture, invasive plants, Kazakhstan, naturalisation

## Abstract

Deliberate cultivation is an important pathway for the introduction of non-native plants, some of which establish populations outside cultivation. We compiled a taxonomically standardised checklist of non-native vascular plants recorded as cultivated in Kazakhstan and compared their regional distributions with the national alien-flora inventory. The checklist contains 286 taxa in 200 genera and 60 families, including 136 ornamental and 88 food taxa. Spontaneous occurrence is documented for 108 taxa (37.8%): 66 casual, 37 naturalised but non-invasive and five invasive. Among cultivation-purpose categories containing more than one taxon, the proportion recorded outside cultivation ranges from 25.0% for industrial plants to 76.5% for forage plants. Five invasive taxa, *Acer negundo*, *Ailanthus altissima*, *Euonymus europaeus*, *Fraxinus excelsior* and *Pilosella aurantiaca*, were assigned Moderate environmental impact ratings in the published national EICAT assessment. The checklist brings together floristic records of cultivation and independently assessed information on spontaneous occurrence and establishment in Kazakhstan.

## 1. Introduction

Agriculture, horticulture, forestry and urban planting have introduced many species beyond their native ranges [1,2,3,4,5,6]. Cultivated plants can supply propagules to surrounding habitats, where some species establish spontaneous populations. These populations may remain casual, become naturalised or become invasive [7,8]. Cultivation and spontaneous establishment therefore require separate floristic records.

Naturalisation is influenced by propagule pressure, residence time, climatic conditions and species traits [9,10,11,12,13,14,15]. The relative importance of these factors varies among plants introduced for ornamental, agricultural, forestry and other uses [5,6,12,13,14,15]. Comparisons between cultivated and spontaneous floras can therefore help to characterise the contribution of different cultivation purposes to the recorded alien flora.

Information on cultivated and alien plants in Central Asia is dispersed among national floras, regional studies, historical inventories and biodiversity records [16,17,18]. The World Cultivated Plants database (WorldCuP) assembles 3050 checklists from 2718 regions in 156 countries, encompassing 102,243 accepted taxa [19]. National floristic inventories complement this global compilation with detailed regional records and taxonomic treatments.

National inventories of alien vascular plants have recently been published for Kyrgyzstan [20,21], Uzbekistan [22] and Kazakhstan [23,24]. The distribution of the vascular flora of Kazakhstan has also been examined in a national synthesis [25]. These studies provide comparative information on floristic composition and spontaneous occurrence, while records of deliberate cultivation require separate compilation.

Cultivation records for Kazakhstan were compiled from Flora of Kazakhstan [26,27,28,29,30,31,32,33,34], Conspectus Florae Asiae Mediae [35,36,37,38,39,40,41,42,43,44,45], Abdulina’s checklist [46] and the regional floristic and taxonomic literature [47,48,49,50,51,52,53,54,55,56,57,58]. Records were assigned to the floristic regions recognised in Flora of Kazakhstan [26]. Spontaneous occurrences were considered separately using the national alien-flora inventory [23,24].

This study documents the taxonomic composition, growth forms, principal uses and regional distribution of cultivated non-native vascular plants in Kazakhstan. It also establishes which taxa have been recorded outside cultivation and summarises their establishment status according to the national alien-flora assessment.

## 2. Results

The checklist contains 286 vascular plant taxa in 200 genera and 60 families. Of these, 277 (96.9%) are angiosperms and nine (3.1%) are gymnosperms. The most species-rich families are Asteraceae (35 taxa; 12.2%), Fabaceae (30; 10.5%), Poaceae (23; 8.0%) and Rosaceae (22; 7.7%). Brassicaceae and Solanaceae each comprise 13 taxa, Cucurbitaceae 11, Apiaceae ten, and Lamiaceae, Malvaceae and Salicaceae seven each. These 11 families account for 178 taxa (62.2%); 24 families are represented by a single taxon.

**Figure 1:**
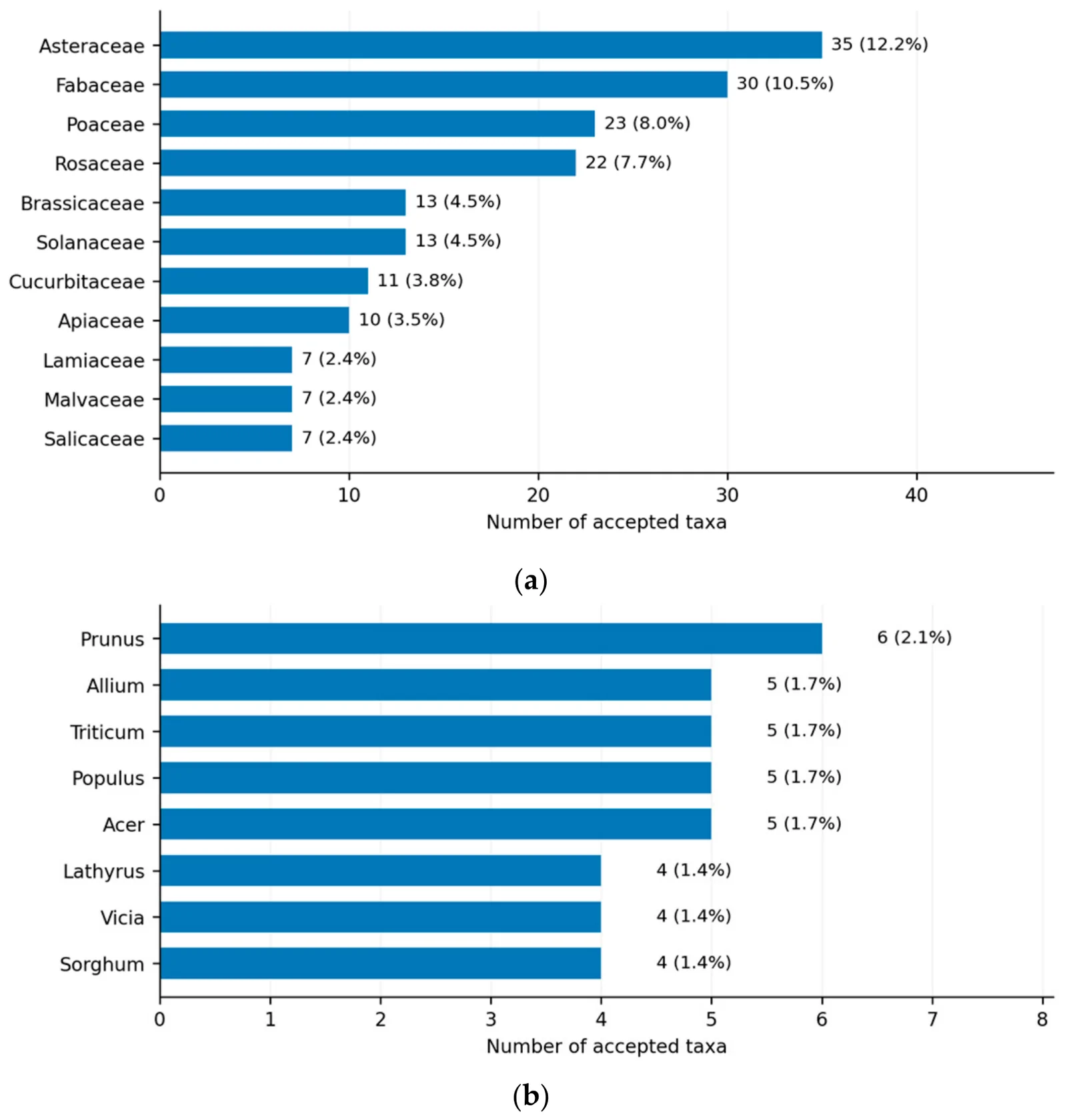
The taxonomic composition of the documented cultivated non-native vascular flora of Kazakhstan. Families with seven or more taxa (**a**) and genera with four or more taxa (**b**) are shown. Each bar is labelled with the number of accepted taxa and its percentage of the full inventory (*n* = 286).

Of the 200 genera, 148 (74.0%) are represented by a single taxon. *Prunus* comprises six taxa; *Acer*, *Allium*, *Populus* and *Triticum* five each; and *Lathyrus*, *Sorghum* and *Vicia* four each (Figure 1).

**Figure 2:**
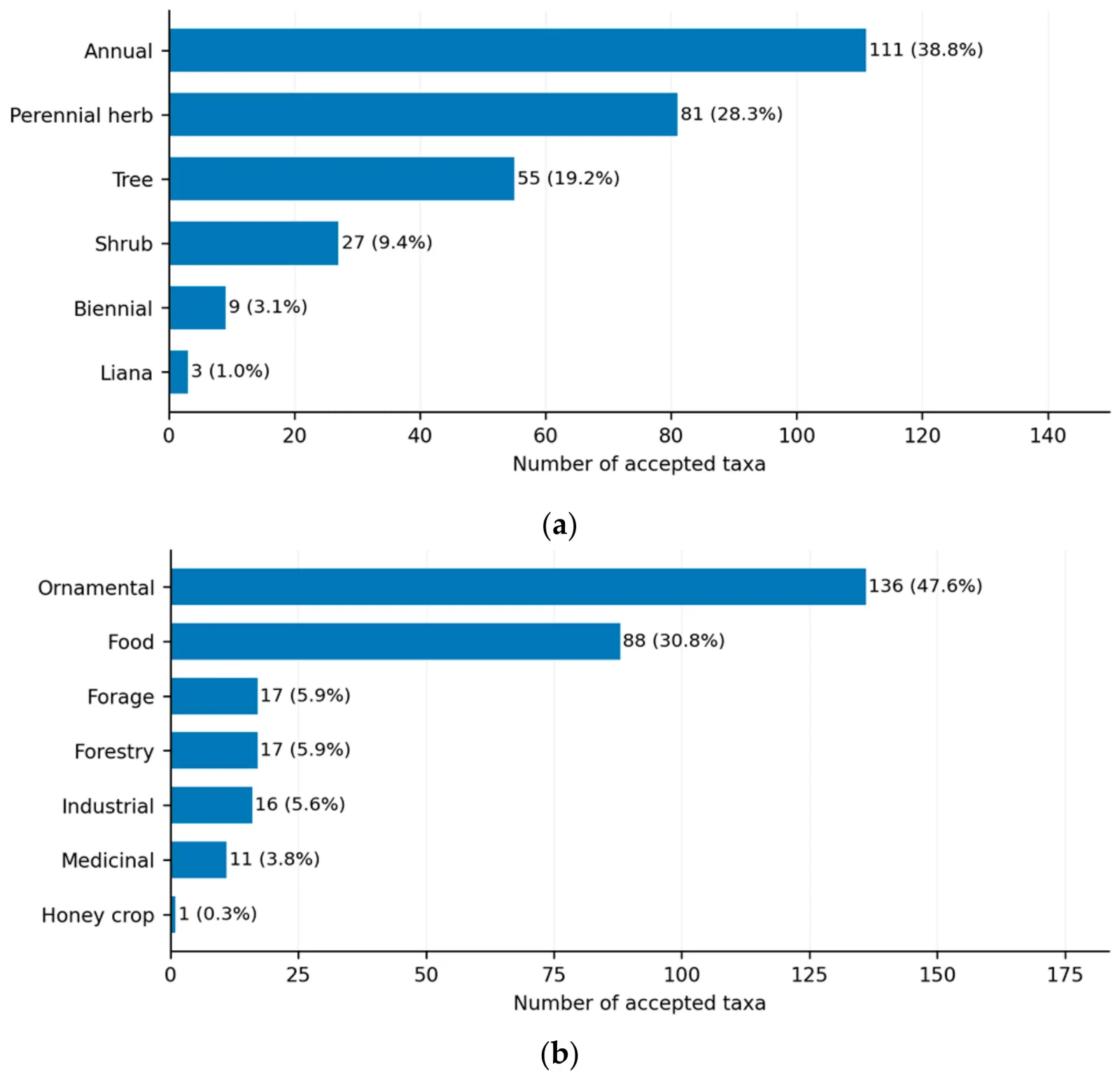
The growth forms (**a**) and principal cultivation purposes (**b**) of the documented cultivated non-native vascular flora of Kazakhstan. The bars show the number of accepted taxa and the corresponding percentage of all 286 accepted taxa; each taxon is assigned to one growth-form and one principal-use category.

Herbaceous plants comprise 201 taxa (70.3%): 111 annuals (38.8%), 81 perennial herbs (28.3%) and nine biennials (3.1%). Woody plants include 55 trees (19.2%) and 27 shrubs (9.4%); three taxa are lianas (1.0%). Ornamental plants form the largest cultivation-purpose group (136 taxa; 47.6%), followed by food plants (88; 30.8%). Forage and forestry plants each account for 17 taxa (5.9%), industrial plants for 16 (5.6%), medicinal plants for 11 (3.8%) and honey crops for one (0.3%) (Figure 2).

Growth-form composition differs among cultivation-purpose groups. Ornamental plants include 48 perennial herbs, 34 annuals, 25 trees and 23 shrubs. Food plants include 52 annuals, 15 perennial herbs and 13 trees. Forage plants comprise ten perennial and seven annual herbs; all 17 forestry taxa are trees. Fourteen of the 16 industrial taxa are annuals.

According to the national alien-flora assessment [23,24], 108 cultivated taxa (37.8%) have been recorded outside cultivation: 66 casual, 37 naturalised but non-invasive and five invasive (Table 1). The invasive taxa are *Acer negundo* L., *Ailanthus altissima* (Mill.) Swingle, *Euonymus europaeus* L., *Fraxinus excelsior* L. and *Pilosella aurantiaca* (L.) F.W.Schultz & Sch.Bip. The naturalised component, including invasive taxa, comprises 42 taxa (14.7% of the checklist). Spontaneous occurrence is documented for 51 of 136 ornamental taxa (37.5%), 24 of 88 food taxa (27.3%), 13 of 17 forage taxa (76.5%) and seven of 11 medicinal taxa (63.6%). By growth form, spontaneous records concern 14 of 27 shrubs (51.9%), 36 of 81 perennial herbs (44.4%), 38 of 111 annuals (34.2%) and 16 of 55 trees (29.1%).

**Figure 3:**
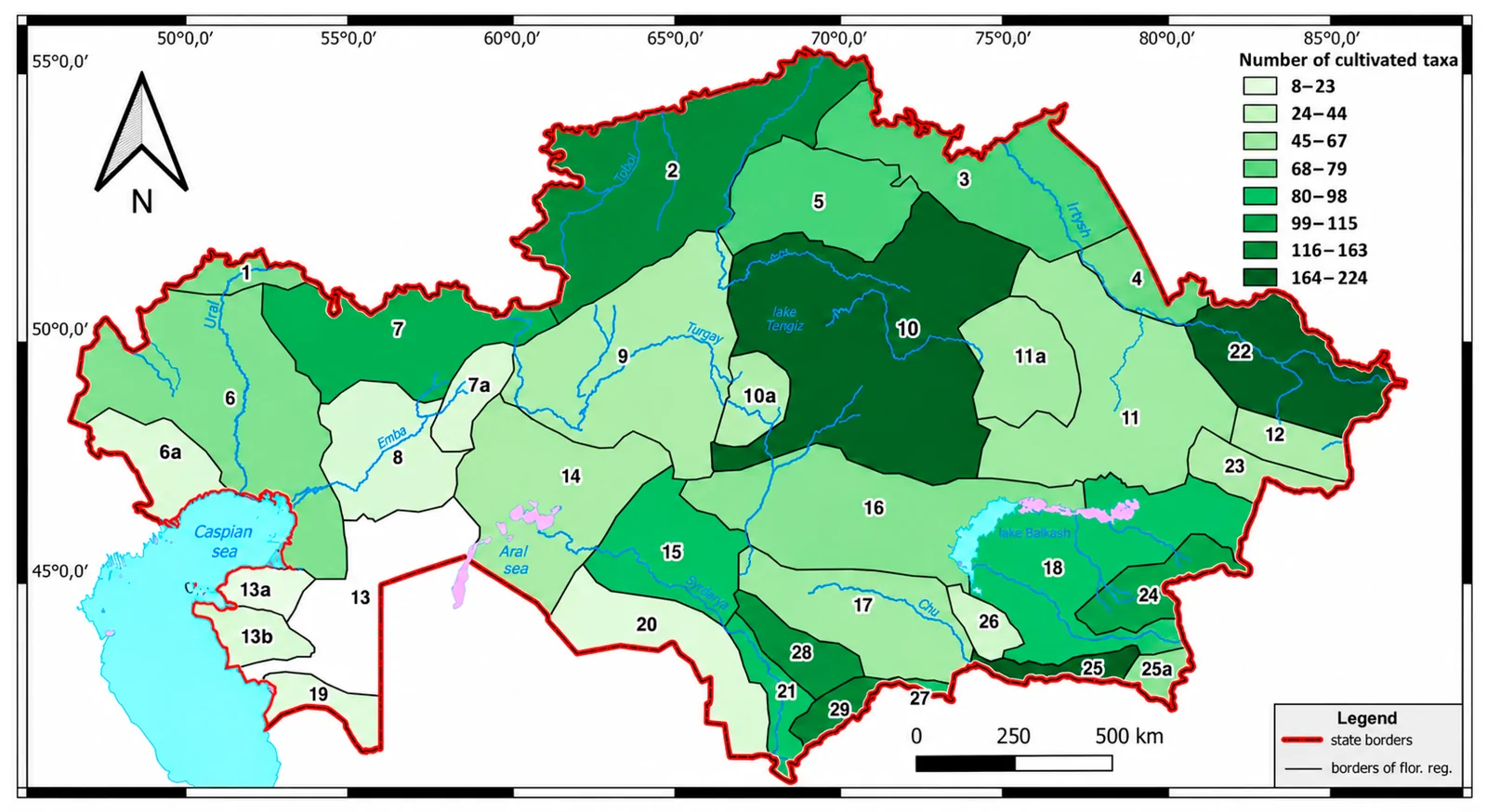
The documented richness of cultivated non-native vascular plant taxa across the floristic regions of Kazakhstan. For codes of floristic regions, see Materials and Methods.

Cultivation is recorded in all 29 floristic regions and seven subregions of Kazakhstan (Figure 3). The largest regional totals occur in the Trans-Ili–Kungey Alatau (region 25; 224 taxa), Altai (22; 163), Western Upland (10; 161), Western Tian Shan (29; 154) and Tobol-Ishim (2; 115). The corresponding numbers of taxa reported outside cultivation are 83, 57, 51, 44 and 40, respectively [23,24].

## 3. Discussion

The 286-taxon checklist comprises records of cultivation assembled from the floristic literature covering different periods. Of these taxa, 108 have also been recorded spontaneously; 42 are classified as naturalised, including five invasive taxa. This comparison describes the recorded cultivated flora and its overlap with the spontaneous alien flora of Kazakhstan.

The global WorldCuP database provides a comparative account of cultivated-plant diversity [19]. For Kazakhstan, the floristic subdivisions used in the national flora allow cultivated and spontaneous records to be compared within the same geographical units [23,24,26]. Principal taxon-specific sources are listed in Supplementary Table S1.

Ornamental plants are the largest cultivation-purpose group (136 taxa), but forage plants have the highest proportion of taxa recorded spontaneously (13 of 17; 76.5%), followed by medicinal plants (seven of 11; 63.6%) among groups represented by more than one taxon (Table 1). The number of ornamental taxa recorded outside cultivation is nevertheless greater (51 taxa). Published comparisons associate naturalisation with cultivation history, propagule supply and biological characteristics [5,6,12,13,14,15,19,59].

Nineteen ornamental taxa (14.0%), nine forage taxa (52.9%) and six food taxa (6.8%) are naturalised or invasive. These proportions differ among the cultivation-purpose groups, but the records do not permit estimation of establishment probabilities because their temporal coverage and sampling effort are heterogeneous.

Spontaneous occurrence is recorded for 14 of 27 shrubs (51.9%), including 12 casual taxa. Among naturalised and invasive plants, annuals and perennial herbs account for 15 taxa each, trees for nine and shrubs for two (Table 1). Growth form is one of several traits associated with naturalisation in comparative studies [12,13,14,60].

The forage plants *Medicago sativa* L., *Lolium perenne* L. and *Lathyrus cicera* L. have both cultivation and spontaneous records in 36, nine and four floristic units, respectively (Supplementary Table S1). All three are classified as naturalised in the national alien-flora assessment [23,24]. In contrast, the compiled sources record *Beta vulgaris* L. under cultivation without a corresponding spontaneous record. These examples identify differences in the geographical documentation of cultivation and spontaneous occurrence, rather than demonstrated dispersal routes.

We follow the definitions of Richardson et al. [7] and Pyšek et al. [61] when distinguishing casual, naturalised and invasive taxa. The reported proportions describe establishment categories documented in the available sources rather than measured rates of transition between these stages.

The regional distribution of cultivated taxa may partly reflect the location of major urban centres and patterns of agricultural and horticultural land use. The Trans-Ili–Kungey Alatau, which includes the Almaty foothill area, has the highest documented richness (224 taxa), including 111 ornamental and 66 food taxa (Supplementary Table S1). The Western Upland, encompassing the Astana area, contains 161 taxa, of which 76 are ornamental and 53 are food plants. In the Western Tian Shan, near Shymkent, food plants (61 taxa) outnumber ornamentals (55), while the Tobol-Ishim region, which includes Kostanay, has 115 cultivated taxa, including 49 ornamental and 43 food plants. The Altai also ranks among the richest regions, with 163 cultivated taxa. These regions also contain numerous taxa recorded outside cultivation [23,24]. The geographical differences may be associated with urban planting, horticulture and agriculture [5,10,11,12,13,14,15,59]. These figures represent the number of documented cultivated taxa; the contributions of individual cities and cultivation practices require further investigation.

Alien-plant richness varies with habitat conditions and land use, as observed in global comparisons [62] and urban surveys in Kazakhstan. In ten cities, Vakhlamova et al. [63] found greater variation in alien-plant richness among habitat types than among cities. The national synthesis by Shaimoldina et al. [25] provides a wider geographical account of vascular plant diversity in Kazakhstan.

Differences in floristic coverage can affect the number of cultivated taxa documented for a region [19]. Regional floras, including Goloskokov’s treatment of the Dzungarian Alatau [58], supply further information on the geographical occurrence of taxa included in the cultivated checklist.

Five taxa included in both the cultivated and spontaneous floras are classified as invasive: *Acer negundo*, *Ailanthus altissima*, *Euonymus europaeus*, *Fraxinus excelsior* and *Pilosella aurantiaca* [23,24]. All five received Moderate (MO) environmental impact ratings in the national EICAT assessment [24]. *Acer negundo* has cultivation and spontaneous records in 33 floristic units; *Euonymus europaeus* is recorded spontaneously in one unit (Supplementary Table S1).

Khusainova et al. [64] examined the local aggressiveness of woody alien plants in southeastern Kazakhstan, including *Acer negundo*. Their field-based observations complement the national EICAT assessment [24], which evaluates documented environmental effects of alien taxa. Establishing an invasion history for individual taxa would require dated occurrence records, while identifying vegetation transformers would require local evidence of changes in plant communities [7]. The available sources do not support a species-specific reconstruction of these processes.

The monitoring of established alien populations can focus on survival, regeneration, spread and effects on native vegetation. Repeated field surveys and permanent plots provide observations of population change and vegetation cover; field-validated remote sensing can assist the mapping of extensive stands [65].

## 4. Materials and Methods

**Figure 4:**
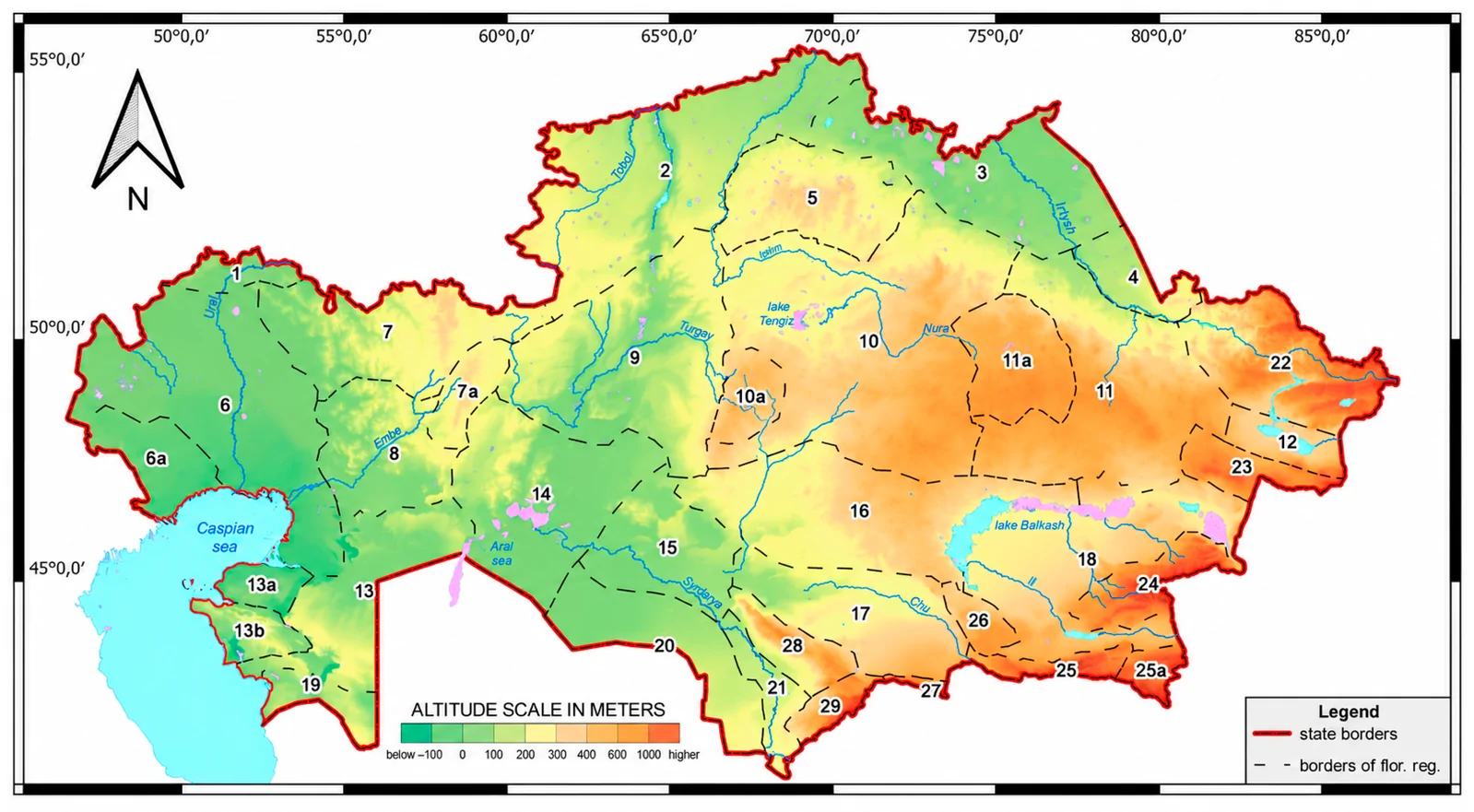
The floristic regions [26] and topography of Kazakhstan: 1—Syrt; 2—Tobol-Ishim; 3—Irtysh; 4—Semipalatinsk pine forest; 5—Kokchetav; 6—Caspian; 6a—Bukeev; 7—Aktobe; 7a—Mugojary; 8—Emba; 9—Turgay; 10—Western Upland; 10a—Ulutau; 11—Eastern Upland; 11a—Karkaraly; 12—Zaysan; 13—Northern Ustyrt; 13a—Buzachi; 13b—Mangyshlak; 14—Aral; 15—Kyzylorda; 16—Betpak-Dala; 17—Moiynkum; 18—Balkhash Alakol; 19—Southern Ustyrt; 20—Kyzylkum; 21—Turkestan; 22—Altai; 23—Tarbagatai; 24—Dzungarian Alatau; 25—Trans-Ili–Kungey Alatau; 25a—Ketmen-Terskey Alatau; 26—Chu-Ili Range; 27—Kyrgyz Alatau; 28—Karatau; 29—Western Tian Shan.

The study encompassed Kazakhstan (approximately 2.72 million km^2^), from forest-steppes and steppes in the north to semi-deserts and deserts in central and western areas and mountains in the east and southeast [66,67,68]. Regional records were assigned to the 29 floristic regions and seven subregions used in Flora of Kazakhstan [26] (Figure 4).

Cultivation records were compiled from Flora of Kazakhstan [26,27,28,29,30,31,32,33,34], Conspectus Florae Asiae Mediae [35,36,37,38,39,40,41,42,43,44,45], Abdulina’s checklist [46] and the regional floristic and taxonomic literature [47,48,49,50,51,52,53,54,55,56,57,58,69,70,71,72,73,74,75,76,77,78,79,80,81,82,83,84,85,86,87,88,89,90,91,92,93,94]. Historical records were retained. Supplementary Table S1 provides the principal cited source for each accepted taxon.

GBIF [95], Plantarium [96] and iNaturalist [97] were consulted manually for locality, identification and regional-occurrence information. Cultivation records and spontaneous occurrences were treated separately; spontaneous distribution and establishment categories were taken from the national alien-flora inventory [23,24].

The checklist includes non-native vascular taxa explicitly recorded as cultivated in Kazakhstan for ornamental, food, forage, forestry, medicinal, industrial or other uses. Published cultivation records were included irrespective of date. Records restricted to ruderal, segetal, roadside or other spontaneous habitats were not treated as evidence of cultivation. Native species cultivated in Kazakhstan were excluded.

The family classification follows APG IV for angiosperms [98] and Christenhusz et al. for gymnosperms [99]. Taxon names and synonyms were standardised using Plants of the World Online [100]; nomenclatural authorship and publication details were checked against IPNI [101] and the Madrid Code [102]. Records referring to the same accepted taxon were combined, retaining their regional information.

The cultivation checklist was compared with the independently compiled national alien-flora checklist [23,24]. The earlier GBIF checklist [103] includes taxa documented only in cultivation, whereas the present checklist also includes cultivated taxa recorded outside cultivation in the national alien-flora inventory [23,24]. Taxa were matched by accepted scientific name, and their principal attributes, regional codes and supporting references are presented in Supplementary Table S1.

Each taxon was assigned one principal cultivation purpose (ornamental, food, forage, forestry, medicinal, industrial or honey crop) and one growth form (annual, biennial, perennial herb, shrub, tree or liana). Where several uses were reported, the principal purpose was selected from the consulted Kazakhstan literature. Establishment status follows the national assessment [23,24] and the terminology of Richardson et al. [7] and Pyšek et al. [61]. Casual, naturalised but non-invasive and invasive taxa were tabulated separately. Published EICAT categories were taken from Alibekov et al. [24]. The reported establishment categories and their proportions were not interpreted as rates of transition from cultivation to naturalisation.

**Figure 5:**
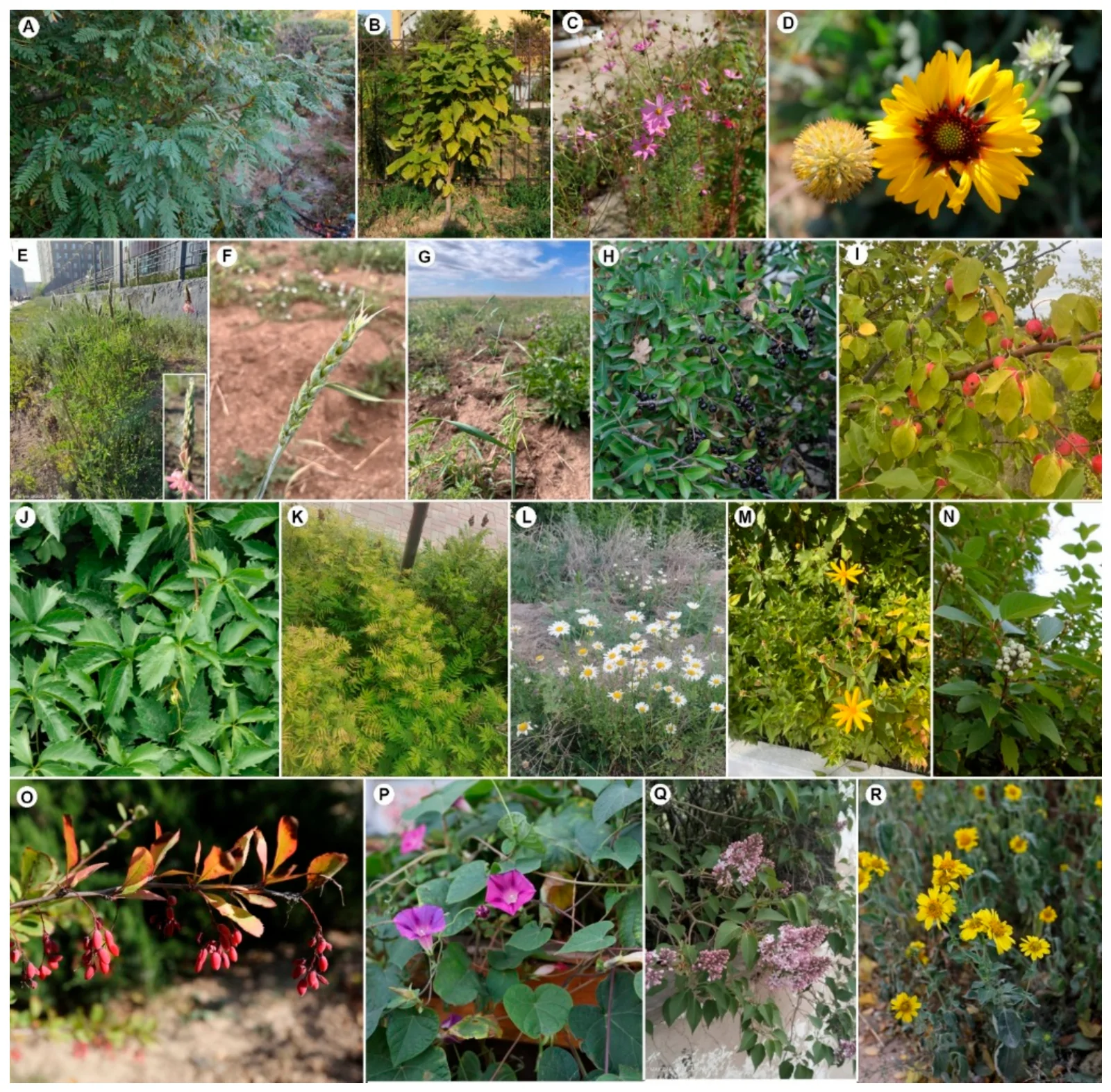
Representative cultivated non-native vascular plant species recorded in Kazakhstan: (**A**) *Amorpha fruticosa* L., (**B**) *Catalpa bignonioides* Walter, (**C**) *Cosmos bipinnatus* Cav., (**D**) *Gaillardia aristata* Pursh, (**E**) *Onobrychis viciifolia* Scop., (**F**) *Triticum aestivum* L., (**G**) *Avena sativa* L., (**H**) *Ligustrum vulgare* L., (**I**) *Malus baccata* (L.) Borkh., (**J**) *Parthenocissus quinquefolia* (L.) Planch., (**K**) *Sorbaria sorbifolia* (L.) A.Braun, (**L**) *Leucanthemum vulgare* Lam., (**M**) *Helianthus tuberosus* L., (**N**) *Symphoricarpos albus* (L.) S.F.Blake, (**O**) *Berberis vulgaris* L., (**P**) *Ipomoea purpurea* (L.) Roth, (**Q**) *Syringa vulgaris* L., (**R**) *Verbesina encelioides* (Cav.) Benth. & Hook.f. ex A.Gray. (Photographs: D. Alibekov and S. Kubentayev (**A**–**P**,**R**); M. Shashakov (**Q**)).

Cultivation and spontaneous occurrence were assigned separate codes for each floristic unit. Cultivation codes were compiled from the floristic literature; spontaneous codes follow the national alien-flora inventory [23,24]. Coordinates in the earlier GBIF cultivated-plant checklist [103] represent regional centroids. The percentages in Figure 1 and Figure 2 are calculated from the full checklist of 286 taxa; those in Table 1 use the number of cultivated taxa in each category. Representative taxa are illustrated in Figure 5.

## 5. Conclusions

The checklist contains 286 cultivated non-native vascular plant taxa in 200 genera and 60 families. Of these, 108 taxa (37.8%) have been recorded outside cultivation, including 42 naturalised taxa. *Acer negundo*, *Ailanthus altissima*, *Euonymus europaeus*, *Fraxinus excelsior* and *Pilosella aurantiaca* are classified as invasive and received Moderate (MO) environmental impact ratings in the national assessment [24]. Ornamental and food plants are the principal cultivation-purpose groups; forage plants have the highest proportion of spontaneous records among groups with more than one taxon. Regional records show the geographical distribution of these taxa across Kazakhstan’s floristic regions.

## Figures and Tables

**Table 1:** The spontaneous occurrence and establishment status of cultivated non-native vascular plants in Kazakhstan by principal cultivation purpose and growth form. Casual, naturalised non-invasive and invasive categories are mutually exclusive; the invasive component is included in the broader naturalised category when totals are combined. Percentages are calculated against cultivated taxa in the corresponding group.

Category	Cultivated Taxa	Casual	Naturalised	Invasive	Recorded Outside Cultivation	Recorded Outside Cultivation, %
*Cultivation purpose*						
Ornamental	136	32	16	3	51	37.5
Food	88	18	6	0	24	27.3
Forage	17	4	9	0	13	76.5
Forestry	17	4	2	2	8	47.1
Industrial	16	3	1	0	4	25.0
Medicinal	11	4	3	0	7	63.6
Honey crop	1	1	0	0	1	100.0
*Growth form*						
Annual	111	23	15	0	38	34.2
Perennial herb	81	21	14	1	36	44.4
Tree	55	7	6	3	16	29.1
Shrub	27	12	1	1	14	51.9
Biennial	9	3	0	0	3	33.3
Liana	3	0	1	0	1	33.3

## Data Availability

The analytical checklist, taxon-level attributes and principal supporting references are provided in Supplementary Table S1. The earlier cultivated-plant checklist is available through GBIF [103]. Spontaneous distribution and establishment data are derived from the national alien-flora checklist and assessment [23,24].

## Supplementary Materials

The following supporting information can be downloaded at: https://www.mdpi.com/article/10.3390/plants15213433/s1, Table S1, Cultivated non-native vascular plants of Kazakhstan (xlsx) provides taxon-level records, principal supporting references and their bibliographic details.
